# Synthesis, Spectroscopic Studies and Keto-Enol Tautomerism of Novel 1,3,4-Thiadiazole Derivative Containing 3-Mercaptobutan-2-one and Quinazolin-4-one Moieties

**DOI:** 10.3390/molecules25225441

**Published:** 2020-11-20

**Authors:** Sewara J. Mohammed, Akam K. Salih, Mohammad Amin M. Rashid, Khalid M. Omer, Karzan A. Abdalkarim

**Affiliations:** 1 Department of Chemistry, College of Science, University of Sulaimani, Qlyasan Street, Kurdistan Regional Government, Sulaimani 46002, Iraq; Akamsalih@univsul.edu.iq (A.K.S.); mohmadamin@univsul.edu.iq (M.A.M.R.); krzanaziz@univsul.edu.iq (K.A.A.); 2 Komar Research Center (KRC), Komar University of Science and Technology, Kurdistan Regional Government, Sulaimani 46002, Iraq

**Keywords:** keto–enol tautomerism, 2-phenyl-4*H*-3,1-benzoxazin-4-one, quinazolin-4-one, 2-amino-1,3,4-thiadiazole-5-thiol, hydrogen bonding, spectroscopic study

## Abstract

In this study, a novel 1,3,4-thiadiazole derivative containing 3-mercaptobutan-2-one and quinazolin-4-one moieties (Compound **3**) is synthesized by the coupling of 2-amino-1,3,4-thiadiazole-5-(3-mercaptobutan-2-one) (Compound **1**) with 2-Phenyl-4H-3,1-benzoxazin-4-one (Compound **2**) in one molecule moiety. Compound **3** is found to exist as two types of intra-molecular hydrogen bonding with keto-enol tautomerism characters, which is further confirmed using FTIR, ^1^H-NMR, ^13^C-NMR, mass spectrometer, and UV-Visible spectra. The ^1^H-NMR and UV-Visible spectra of Compound **3** are investigated in different solvents such as methanol, chloroform, and DMSO. Compound **3** exhibits keto-enol tautomeric forms in solvents with different percentage ratios depending on the solvent polarity. The ^1^H-NMR and UV-Visible spectral results show that Compound **3** favors the keto over the enol form in polar aprotic solvents such as DMSO and the enol over the keto form in non-polar solvents such as chloroform. The ^13^C-NMR spectrum gives two singles at δ 204.5 ppm, due to ketonic carbon, and δ 155.5 ppm, due to enolic carbon, confirming the keto-enol tautomerism of Compound **3**. Furthermore, the molecular ion at *m/z* 43 and *m/z* 407 in the mass spectrum of Compound **3** and fragmentation mechanisms proposed reveal the existence of the keto and enol forms, respectively.

## 1. Introduction

Tautomerism is pervasive in organic chemistry, which allows different properties to coexist in the same molecule. Tautomerisms are structural isomers that differ from one another on the basis of the position of proton(s) and double bonds; the carbon skeleton of the structure is unchanged [1]. It was first described by Laarin in 1886 [2].Keto-enol tautomerism in β-ketoester [3,4,5], β-diketones[6,7,8,9], β-ketonitrile [10], β-ketoamide [11,12,13], ortho-hydroxy Schiff bases [14,15,16,17], Azo dyes [18], alpha-ketophosphonates [19], and the 1,3,4-thiadiazole group with a 2,4-hydroxyphenyl function [20] have been studied using different spectroscopic techniques. Similarly, those compounds of type β-keto-enol derivatives containing heterocyclic moieties such as 4-hydroxycoumarin [21], Imidazoline derivatives [22], indol [23], and pyrazole, pyridine, thiophene, or furan [24] can exist as a keto-enol tautomerism. In most cases, hydrogen transfer is the main reason for the keto-enol formation. The keto-enol are formed also in heterocyclic compounds containing the carbonyl group with acidic methylene protons connected to the heteroatom or to electro-withdrawing species, such as thiazolidine-2,4-dione [25], phenacyl bezoyl pyridinium [26], and 2-phenacyl benzoxazoles [27].

The heterocyclic compounds that bear the keto-enol tautomerism play an important role in biological reactions.They were also used as intermediates to synthesize several other new heterocyclic compounds. The role of this biological effective character arises from their properties related to drugs for the treatment of many different diseases. The β-keto-enol derivatives containing the 1,3,4-thiadiazole group with 2,4-hydroxyphenyl function [20] show many biological effects including antitumor [28], antifungal [29], antibacterial [29], anti-immflammatory [30,31], anticonvulsant [32], antiviral [33], antituberculosis [34], antihypertensive [35], antidepressant activity [36], and anti-androgens [37]. Therefore, a deep understanding of the tautomeric mechanism is very significant for medicinal chemistry perspective.

The aim of this work involves the synthesis of a novel 1,3,4-thiadiazole derivative containing 3-mercaptobutan-2-one and quinazolin-4-one moieties in one molecule (Compound **3**). Experimental investigations are carried out in various solvents to determine the influence of polar and non-polar solvents on the keto-enol tautomerism equilibria. A mechanism of the tautomerism is proposed on the basis of the experimental results.

## 2. Experimental Section

### 2.1. Materials and Characterization

All chemicals were obtained from Merck Company. Melting points were taken using an (OptiMelt) automated melting point instrument (Sunnyvale, CA, USA). The reactions were monitored with TLC using silica gel on TLC plates DC-Glasplatten-Kieselgel (Radnor, PA, USA). A UV lamp was used for the visualization of the TLC spots. The IR spectra were recorded in KBr pellets (v_max_ in cm^−1^) on a Perkin-Elmer FTIR spectrophotometer (Waltham, MA, USA). 1H-NMR and 13C-NMR spectra were recorded on Bruker DRX-500MHz (Billerica, MA, USA), NMR reported in ppm scale. Mass spectra were obtained using Agilent Technology (Santa Clara, CA, USA) and MS Model 5973 Network Mass selective detector.

### 2.2. Synthesis of the Compounds (**1**, **2**, and **3**)

#### 2.2.1. Synthesis of 2-Amino-1,3,4-Thiadiazole-5-(3-Mercaptobutan-2-One) (Compound **1**)

To a stirring solution of 5-amino-1,3,4-thiadiazole-2-thiol (0.01 mol, 1.33 g) and potassium carbonate (0.01 mol, 1.61 g) in 8 mL water, 3-chloro-2-butanone (0.01 mol, 1.07 g, 1.02 mL) was added. The mixture was left to stir for 1 h at room temperature. After the completion of the reaction and being checked by single-spot TLC (eluent: ethyl acetate: n-hexane (1.5: 0.5)), the white solid was filtered and purified by recrystallization from ethanol with n-hexane (yield: 86.8%; m.p.128–129 °C; Rf = 0.17). IR (KBr) v_max_(cm^−1^): 3247and 3108 (NH**_2_**); 1703 (C=O ketone); 1630 (C=N); 1397 (C-O); 1134 (C-N); 687 (C-S). **^1^**H-NMR (500 MHz, DMSO-*d_6_*); δ 7.46 (s, 2H, -NH_2_); 4.18 (q, 1H, CH_3_-CH, *J* = 7 Hz); 2.28 (s, 3H, O=C-CH_3_); 1.35 (d, 3H, CH-CH_3_, *J* = 7 Hz). **^13^**C-NMR (500 MHz, DMSO-*d_6_*): δ 204.8 (1C, C=O); 171.5 (1C, C=N); 171.4 (1C, C=N); 146.4 (1C, S-C-S); 146.3 (1C, N-C-S); 52 (1C, CH3-CH); 27.7 (1C, O=C-CH3); 16.6 (1C, CH-CH3). MS (*m/z*): 203 [M^+^] for C_6_H_9_N_3_OS_2_ (75%); 161 [C_5_H_7_NOS_2_^+^] (100%); 146 [C_4_H_4_NOS_2_^+^] (30%); 128 [C_4_H_2_NS_2_^+^] (85%); 60 [C_2_H_4_S ^+^] (48.75%); 43 [C_2_H_3_O^+^] (63.75%).

#### 2.2.2. Synthesis of 2-Phenyl-4H-3,1-Benzoxazin-4-One (Compound **2**)

Compound **2** was synthesized following a procedure described in the literature [38]. Benzoyl chloride (0.05 mol, 7.03 g, 5.81 mL) was added to a stirring solution of anthracitic acid (0.05 mol, 6.86 g) in pyridine (25 mL), dropwise, maintaining a temperature between 0–5 °C for 1 h. Then, the reaction mixture was left to stir at room temperature for 2 h until a solid product was formed and the completion of the reaction was checked with TLC (eluent: ethyl acetate: n-hexane (1.5: 0.5)). The reaction mixture then was neutralized by adding saturated sodium bicarbonate solution. The formed yellow product was filtered, then washed with water, and recrystallized from ethanol, (yield: 81%; m.p.122–123 °C; Rf = 0.68).

#### 2.2.3. Synthesis of 3-[5-(3-Mercaptobutan-2-One)-1,3,4-Thiadiazol]-2-Phenylquinazolin-4(3H)-One (Compound **3**)

To a solution of Compound 2 (7.50 mmol, 1.80 g) in dry pyridine (15 mL), Compound **1** (11.25 mmol, 2.30 g) was added slowly with constant stirring for 10 min. Then, the reaction mixture was left to reflux for 9 h. After reaction was completed and checked by single-spot TLC (eluent: ethyl acetate: n-hexane 1.5: 0.5), the hot solution was poured in to a beaker containing 100 g of crushed ice and 5 mL of concentrated hydrochloric acid. The light yellow solid product was separated, then filtered, washed with hot ethanol, and then dried to yield the compound product (yield: 69%; m.p.198 °C; Rf= 0.48). IR (KBr) v_max_(cm^−1^): 3435 (OH); 3316 (N-H asym); 3255 (N-H sym.); 2928 (S…H-O); 1707 (C=O of aliphatic); 1687 (C=C enol form); 1655 (C=O lactam); 1606 (C=C aromatic); 1524 (C=N); 1300 (C-O); 758 (mono-substituted aromatic ring); 693 (C-S). **^1^**H-NMR(500 MHz, DMSO-*d_6_*) δ:13.26 (s, 1H, NH zwitterion); 10.95 (s, 1H, OH enol); 8.09 (s, 1H, aromatic); 7.93–7.92 (d, 3H, aromatic, *J* = 7.5 Hz); 7.70–7.47 (m, 4H, aromatic); 7.33–7.31 (t, 1H, aromatic, *J* = 8 Hz); 4.52–4.47 (q, 1H, CH_3_-CH, *J1* = 7.5 Hz, *J2* = 7Hz); 2.31 (s, 3H, O=C-CH_3_); 1.45-1.44 (d, 3H, CH-CH_3_, *J* = 7 Hz). **^13^**C-NMR (500 MHz, DMSO-*d_6_*) δ:204.5 (O=C-CH_3_); 165.1 (O=C-N lactam); 155.5 (1C, C=C-OH, enol); 137.9 (1C, N=C-N); 134.4, 132.8, 132, 129.5, 128.7, 127.4, 123.9, 123.5, 122.9 (aromatic carbon); 51.6 (1C, CHCH); 27.2 (O=C-CH_3_); 16.4 (1C, CH-CH_3_). Dept 135 (500 MHz, DMSO-*d_6_*) δ:132.5, 131.8, 129.3, 128.5, 127.2, 123.7, 122.7, 51.3, 27, 16.2. Deptq (500 MHz, DMSO-*d_6_*) δ: 137.9, 134.4, 123.5 (carbon not connected to hydrogen); 132.8, 132, 129.5, 128.7, 127.4, 123.9, 122.9 (aromatic carbon connected to hydrogen (Ar-H); 51.6, 27.2, 16.4 (aliphatic carbon connected to hydrogen (C-H). MS (*m/z*): 407 [M^+^-H] for C_20_H_16_N_4_O_2_S_2_ (4.8%);224 [C_12_H_8_N_4_O^+^] (100%); 203 [C_9_H_5_N_3_OS^+^] (20.8%); 179 [C_10_H_11_OS^+^] (12%); 161 [C_5_H_7_NOS_2_^+^] (28%); 146 [C_4_H_4_NOS_2_^+^] (25.6%); 120 [C_8_H_8_O^+^] (16.8%); 105 [C_7_H_5_O^+^] (87.2%); 77 [C_6_H_5_^+^] (64%); 60 [C_2_H_4_S^+^] (6.4%); 43 [C_2_H_3_O^+^] (35.2%).

## 3. Results and Discussion

**Scheme 1:**
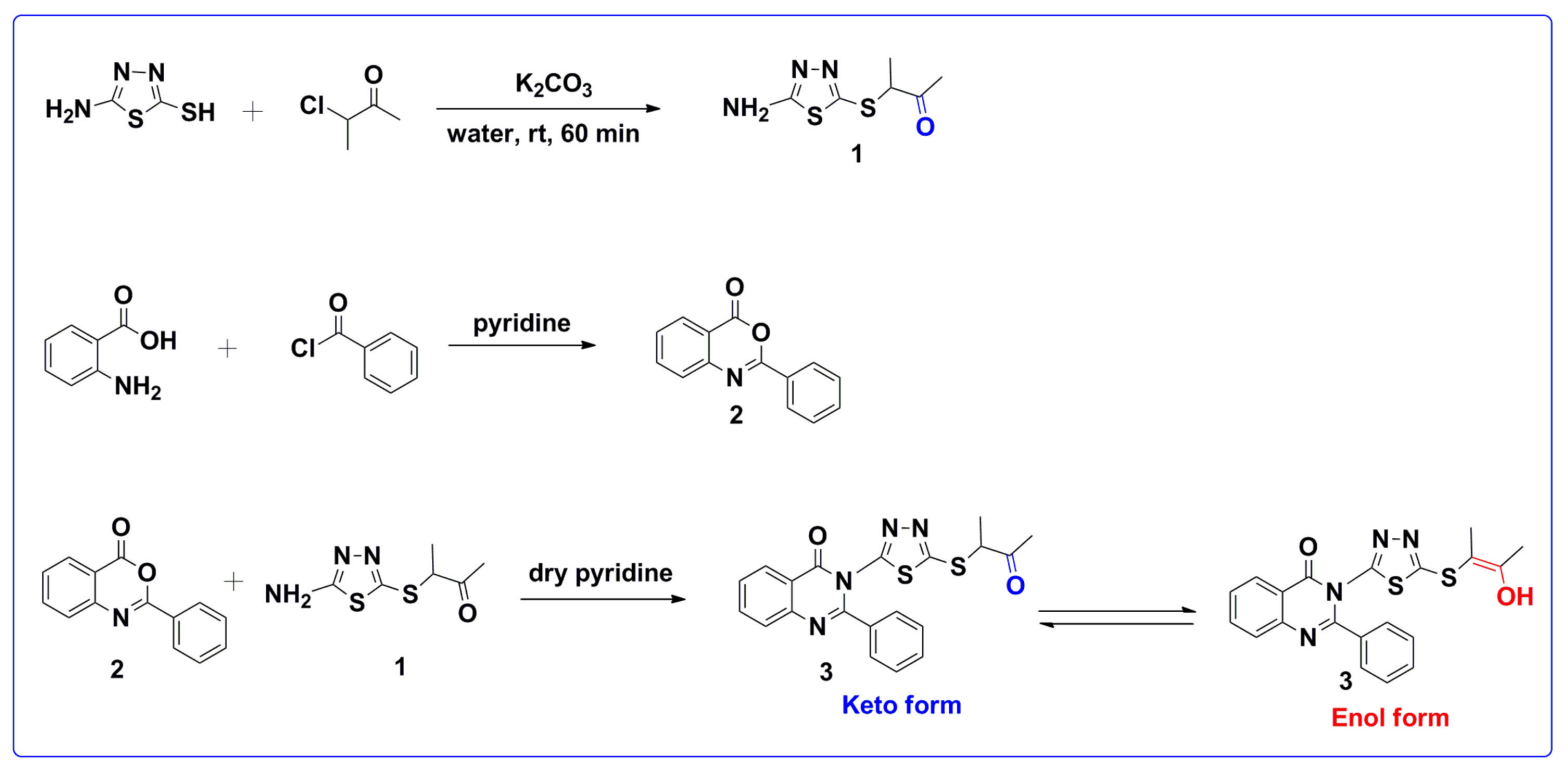
Synthetic route of Compounds (**1**, **2**, and **3**).

Compound **3** was synthesized from the coupling of an intermediate compound of 2-amino-1,3,4-thiadiazole-5-(3-mercaptobutan-2-one) (Compound **1**) with 2-Phenyl-4H-3,1-benzoxazin-4-one (Compound **2**), as shown in Scheme 1. The intermediate Compound **1** was obtained from the reaction of 2-amino-1,3,4-thiadiazole-5-thiol with 3-chloro-2-butanone in the presence of potassium carbonate, using water as a green solvent. The structure of Compounds **1** and **3** were determined on the basis of their FTIR, ^1^H-NMR, ^13^C-NMR, and mass spectra.

Compound **3** shows intra-molecular hydrogen bonding together with keto-enol tautomerism, which was determined using FTIR, ^1^H-NMR, ^13^C-NMR, and UV-Visible spectroscopy.

### 3.1. Structural Characterization (FTIR, ^1^H-NMR, ^13^C-NMR, Mass, and UV-Visible)

#### 3.1.1. FTIR Study

**Figure 1:**
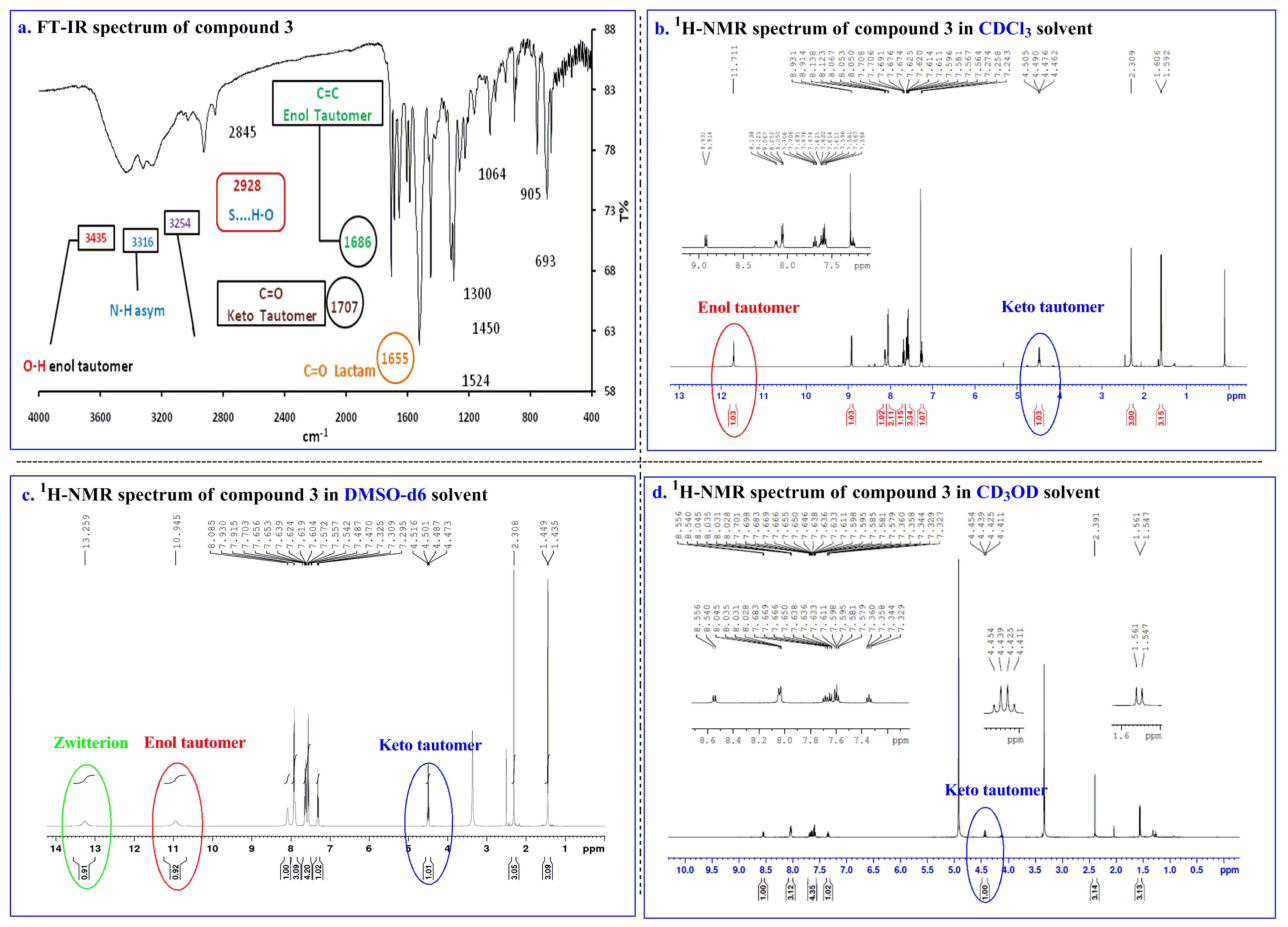
(**a**) FTIR spectrum of Compound **3**. (**b**–**d**) ^1^H-NMR spectra of Compound **3** in different solvents.

**Scheme 2:**
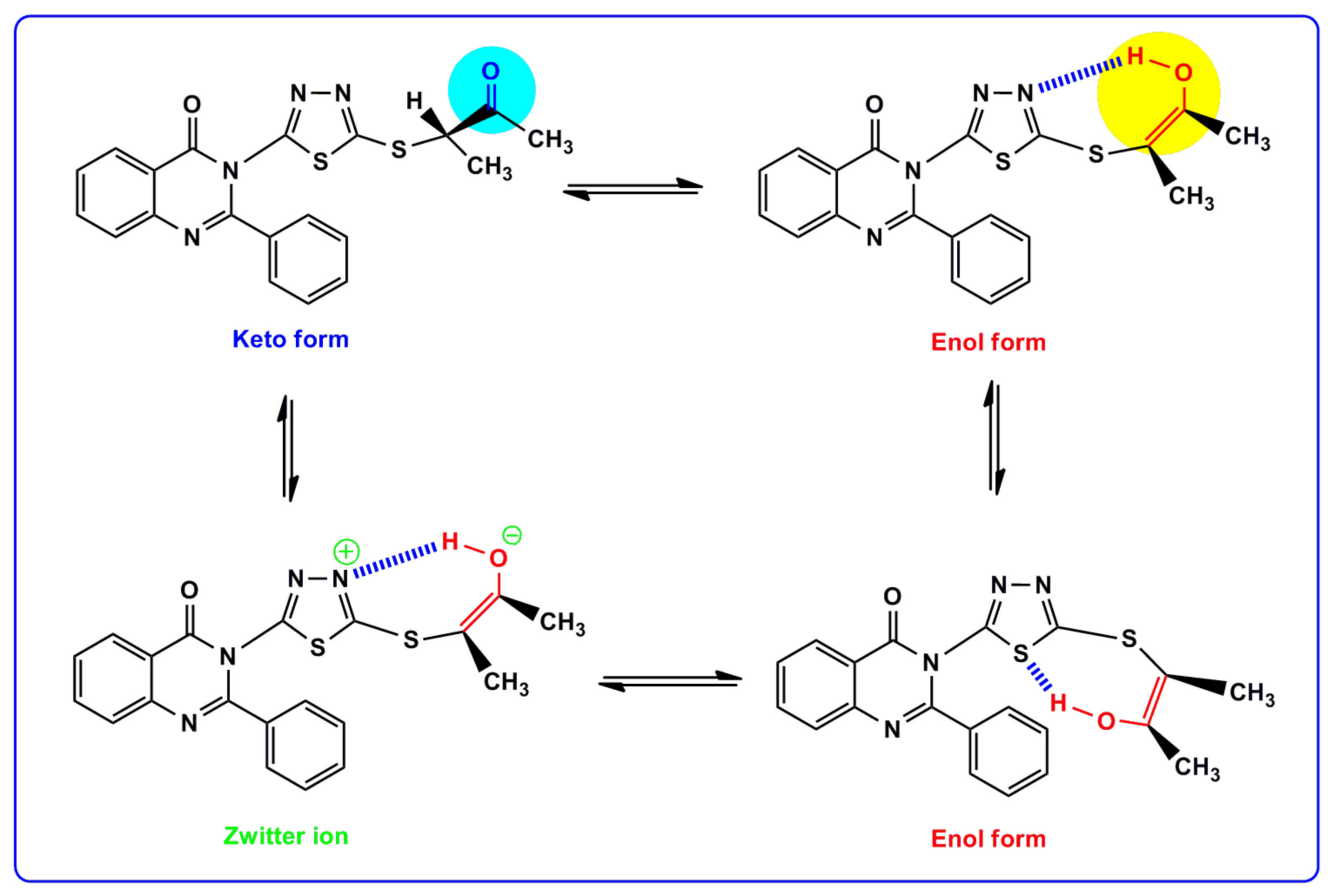
Keto-enol form of Compound **3**.

The FTIR spectrum shown in Figure 1a confirms the proposed structure of the keto-enol form (Scheme 2). The FTIR spectrum showed medium and broad absorption bands in the region 3436–3255 cm^−1^ and a medium sharp band at 2928 cm^−1^, which were assigned to the enol tautomer character that was probability involved in the intra-molecular hydrogen bonding. Additionally, strong absorption bands were observed in the region 1707–1655 cm^−1^, which we assigned to the presence of the keto character. The broad stretching vibration absorption band at 3436 cm^−1^ could be assigned to the O-H group of enol, which indicates the involvement in intra-molecular hydrogen bonding with nitrogen atom of 1,3,4-thiadiazole unit (N…H-O) [39,40]. While the medium sharp band at 2928 cm^−1^ was assigned to (S…H-O) intra-molecular hydrogen bonding [41]. Whereas two medium broad bands were observed at 3316 and 3255 cm^−1^, which we referred to the N-H that was involved in intra-molecular hydrogen bonding (N-H…..O) [40,42]. Then, three other absorption bands at 1707, 1655, and 1686 cm^−1^ were observed and these bands were assigned to the stretching vibrations of carbonyl of aliphatic ketone, lactam, and C=C groups in both the keto and enol forms [43]. Therefore, the FTIR data of Compound **3** shows the existence of two possible tautomers, such as the ketoand enol forms, which are obtained by aninterchanging behavior through a proton transfer reaction between the keto and enol tautomers (Scheme 2).

There are many factors that contribute to the enolization of Compound **3**;

First: the acidity of α-hydrogen between the carbonyl and sulfur atom.

Second: the presence of a conjugated double bond with aintra-molecular hydrogen bond that helps to stabilize the enol form [44].

Third: the electron-accepting character of the sulfur atom adjacent to 1,3,4-thiadiazol moiety that stabilizes the enol form by electron delocalization [45].

On the contrary, the FTIR and ^1^H-NMR spectra of Compound **1,** that possesses the same functional group of 3-mercaptobutan-2-one similar to Compound **3,** were recorded, and a signal that was assigned to the proton for the OH of the enol form was not detected, presumably due to the low electronic reactivity and high basicity of Compound **1** that makes the α-hydrogen present as a very low acidic character. From these results, one can conclude that Compound **1** is present only as the keto form. However, the occurrence of the quinazolin-4-one moiety in Compound **3** with a moderately high polar surface area affects the electronic property of the complete molecule and increases the acidity of α-hydrogen, which helps to generate the keto-enol tautomerization. Further evidence for the confirmation of the existence of Compound **3** as keto-enol tautomeric forms is supported by ^1^H-NMR spectroscopy analysis data in different solvents.

#### 3.1.2. H-NMR Study

The ^1^H-NMR of Compound **3** was recorded at room temperature in different solvents such as CDCl_3_, DMSO-*d_6_*_,_ and CD_3_OD. Figure 1b–d represent the changes in the spectral characteristics of Compound **3** by changing the solvent from non-polar to polar organic solvents. The main absorption peaks of this compound are tabulated in Table 1.

The ^1^H-NMR of Compound **3** in CDCl_3_ at room temperature shows a relatively broad signal at 11.71 ppm, which was assigned to the hydroxyl proton involved in the intra-molecular (O-H…N) hydrogen bonding [46], and another signal of the proton at α-position was observed at 4.46–4.51 ppm, which was attributed to methine proton (CH group) in keto form. The enol form was dominated in CDCl_3_, this means that Compound **3** in CDCl_3_ undergoes enolization to produce a keto-enol tautomerism in a non-polar solvent. This process occurs because α-hydrogen adjacent to the carbonyl group is slightly acidic. Whereas the ^1^H-NMR spectrum of this compound in the deuterium DMSO solvent shows two significant signals: first, the small broad one of the hydroxyl proton at 10.95 ppm that is due to the strong intra-molecular (O-H….N) hydrogen bonding and, second, another remarkable small broad peak downfield at 13.26 ppm that was integrated for one proton and related to the existence of intra-molecular (O…H-N) hydrogen bonding [47]. On the basis of this result, it was confirmed that the Compound **3** was obtained as a keto-enol tautomeric form possibly through the zwitterion [48] form with a cationic nitrogen atom of the 1,3,4-thiadiazole unit and anionic enolate groups that completed a five-membered pseudo cycle via an intra-molecular (N-H….O) hydrogen bond. The positions of the hydroxyl proton and the N-H proton of Compound **3** in the DMSO solvent in the presence of D_2_O showed a deuterium exchange between active protons and the D_2_O solvent through the disappearance of any signal, due to OH and N-H protons at 10.95 ppm and 13.26 ppm, respectively, upon being shaken with D_2_O. In the DMSO-*d_6_* solvent, the keto form of Compound **3** predominated over the enol form. Increasing the solvent polarity caused the increase in the proportion of the keto form. This effect can be explained by considering that DMSO-*d_6_* is a hydrogen bond acceptor solvent and it competes with the carbonyl group in the establishment of the hydrogen bond. The destabilization must be greater in the enol tautomer and this causes the shift in the equilibrium toward the keto form. While the ^1^H-NMR spectrum of Compound **3** in a protic polar solvent like CD_3_OD was recorded, a signal was not detected, due to OH and NH protons at 10.95 ppm and 13.26 ppm, respectively. This observation was attributed to some proton-deuterium exchange that must have occurred between the active proton of each hydroxyl or NH group of Compound **3** and the solvent CD_3_OD. Different solvents have an effect on the shifting of the tautomerism, the relative abundance of tautomeric forms is determined by the integration of CH and OH groups. The spectra data (Table 2) demonstrates that tautomeric equilibrium depends on the solvent and an increase in solvent polarity like deuterium DMSO increases the proportion of the keto form compared to chloroform. The change of the solvent polarity leads to a change in the hydrogen bonding character and thus causesa change in the equilibrium of the tautomerism. The ^1^H-NMR spectra show that the relative percentage yield of the keto form is more than the enol form in dimethyl sulfoxide solution compared to the use of chloroform as a solvent, and this difference in the quantitative keto-enol equilibrium refers to a more polar property of the keto form than the enol form and, hence, is more stable in a polar solvent such as dimethyl sulfoxide compared to the nonpolar solvent chloroform. Thus, the position of the keto-enol equilibrium strongly depends on the electronic character and the nature of the solvent [49]. The cis-enol form is stabilized by a strong intra-molecular hydrogen bond with its double bond character [50].The hydrogen bond formation in enol leads to an enhancement of the resonance conjugation of the π-electrons which causes a marked tendency for the equalization of the bond order of the valence bonds in the result and possesses a structure of the pseudo cyclic-membered chelated ring [51], which is more favorable in the non-polar solvent. The percentage of its tautomerism was calculated from the ratio of enol to keto and is related to the equilibrium constant as shown in Equation (1):

(1) $$Kc=\frac{\%\;\mathrm{enol}}{\%\;\mathrm{keto}}$$

The equilibrium constant depends on the solvents which are related to the solvent dielectric constant versus the tautomer polarity stability. The relative strengths of these ^1^H-NMR signals will be proportional to the concentration of each different form. Thus, the equilibrium constant for each spectrum can be determined by measuring the integrated ^1^H-NMR signals for the enol hydroxyl and keto methine proton (Table 2), using the following relationship in Equation (2):

(2) $$Kc=\frac{\%\;\mathrm{Area}\;\mathrm{methin}\;\mathrm{signal}}{\%\;\mathrm{Area}\;\mathrm{hydroxyl}\;\mathrm{signal}}$$

#### 3.1.3. C-NMR Study

**Figure 2:**
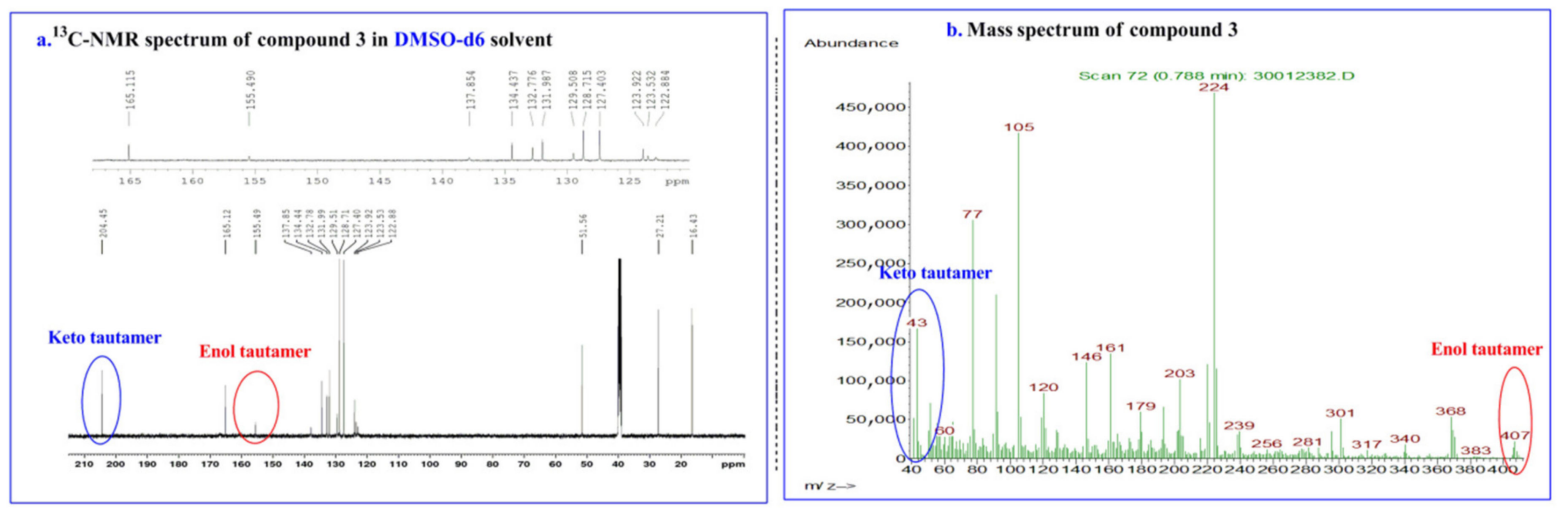
(**a**) ^13^C-NMR spectrum of compound **3** in DMSO-*d_6_*. (**b**) Mass spectrum of Compound **3**.

The ^13^C-NMR spectrum of Compound **3** in DMSO solvent (Figure 2a) shows three absorption bands at 16.2, 27.2, and 51.6 ppm which refer to saturated carbons of type CH_3_-CH, CH_3_-C=O, and CH_3_-CH, respectively. Nine signals between 122.9–134.4 ppm were assigned to twelve aromatic carbons together with one carbon of cyclic quinazolin-4-one ring and one carbon of double bond of enol group (C=C-OH). Two signals were assigned to C=N and (C=C-OH) enol carbons atoms and appeared at 137.9 ppm and 155.5 ppm, respectively [52]. In addition, another two significance absorption bands were observed at 165.1 and 204.5 ppm and were assigned to two carbons of carbonyl carbon of lactam moiety and aliphatic ketone, respectively [53]. These assignments are consistent with the existence of Compound **3** as keto-enol tautomer forms (Table 3)**.**

#### 3.1.4. Mass Spectroscopy Study

**Scheme 3:**
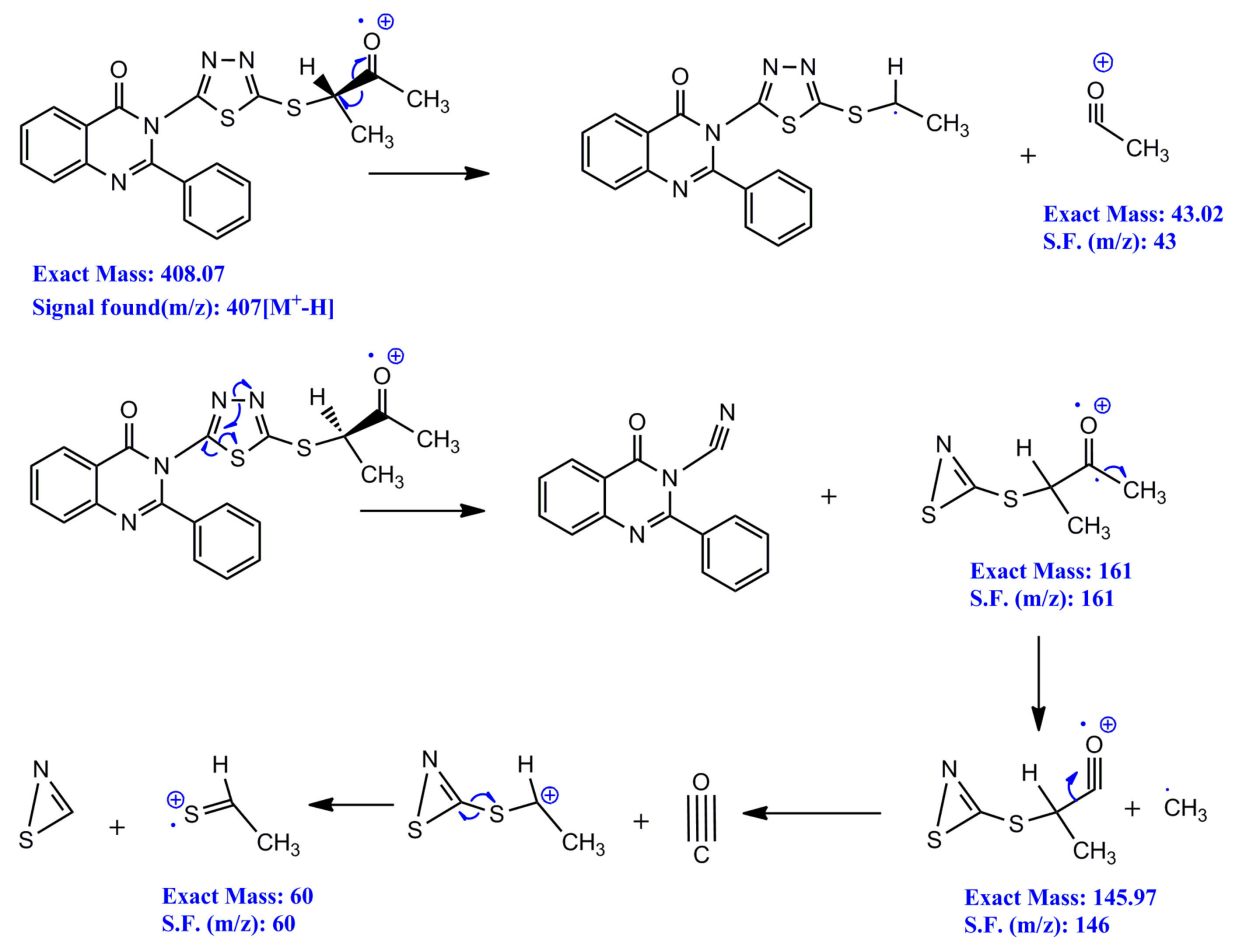
Suggested fragmentation of keto form Compound **3.**

**Scheme 4:**
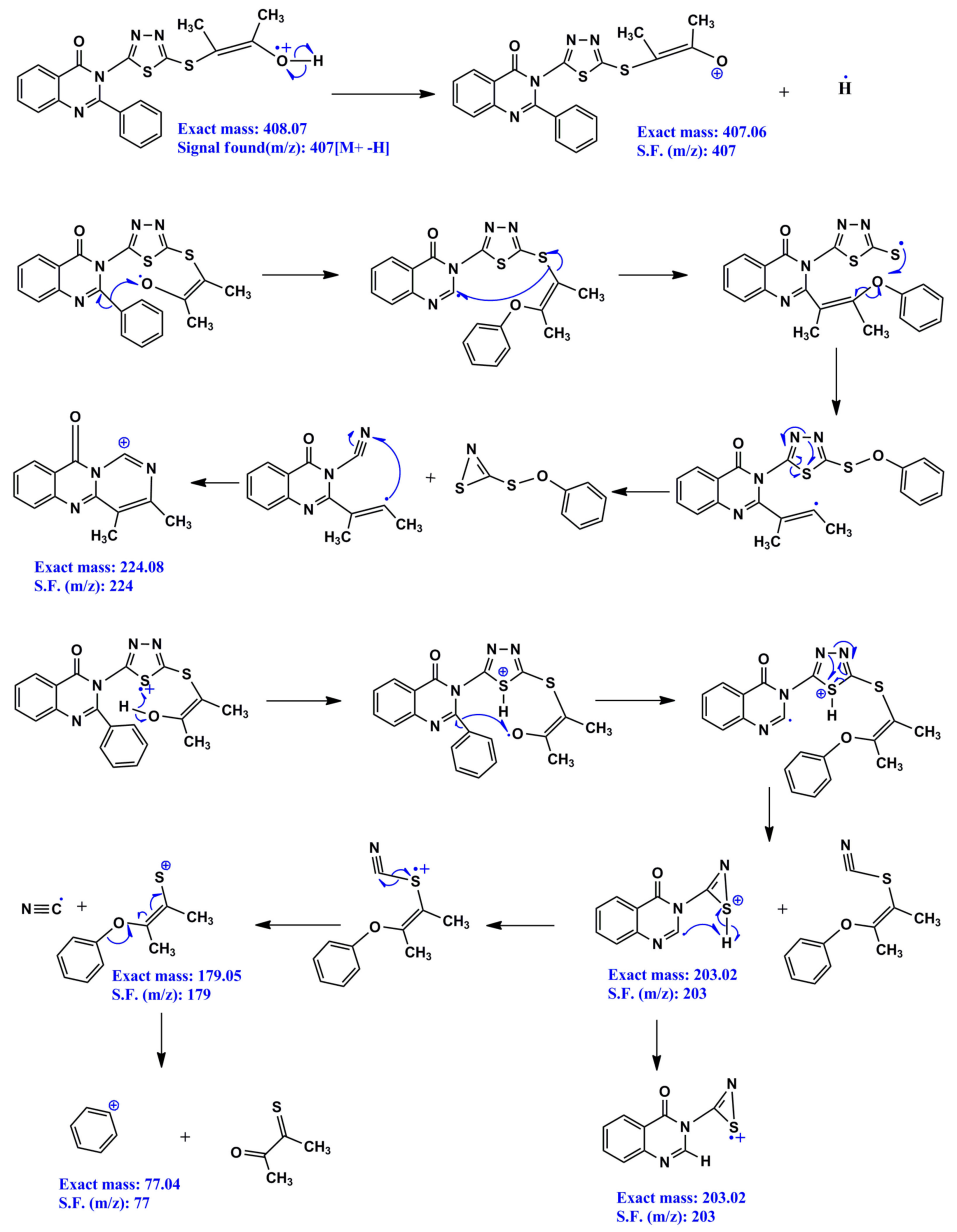
Suggested fragmentation of enol form Compound **3**.

The mass spectrum of Compound **3** is shown in Figure 2b with proposal fragmentation patterns, and the relative intensities of the characteristic ion peaks in mass spectrum are given in Table 4**.** From this spectrum, it was observed that the keto and enol forms of Compound **3** possess completely different fragmentation patterns. The basic signal at *m/z* = 407 was formed with very low intensity abundance with the loss of one hydrogen atom (m-H^+^). This peak can be attributed to the enolic tautomer [54]. The processes of formation of the fragments of Compound **3** are shown in Scheme 3 and Scheme 4. The keto-enol tautomerization of Compound **3** was established on the basis of the comparison of mass spectrometric fragmentation patterns of Compound **3** with fragmentation patterns of Compound **1** (Table 5). The mass spectrum of Compound **3** shows a peak at *m/z* 43 which was raised from the fragmentation pathway. Similarly, the mass spectrum of Compound 1 shows a peak at *m/z* 43 that can be explained with that it is following the same fragmentation pathway. The ion with proposal formula CH_3_CO^+^ can be assigned to the keto tautomer [55]. In addition, some other fragmentation peaks at *m/z* 161, 146, and 60 are observed which can exist only in the keto tautomer by analogy with the fragmentation of keto tautomer of Compound **3** (Scheme 3)**.** This means that the Compound **3**that is studied existed in the keto form. On the other hand, the appearance of the fragment ions at *m/z* 77 is assigned to C_6_H_5_^+^ molecular ion, and two significant peaks at *m/z* 105 and 120 are assigned to C_6_H_5_C^+^ and [C_6_H_5_CO-CH_3_]^+^ molecular ions respectively indicate for existence of the enol form. More molecular ion peaks at *m/z* 224, 203, 179, 120, 105, 77 with different relative abundance were detected from the spectrum of Compound **3** (Scheme 4). These signals and the molecular ion with a loss of a proton are evidence for the presence of the enol tautomer. Finally, in the results, all the fragmentations of Compound **3** gave evidence of the existence of keto-enol tautomers.

#### 3.1.5. UV-Visible Study

**Figure 3:**
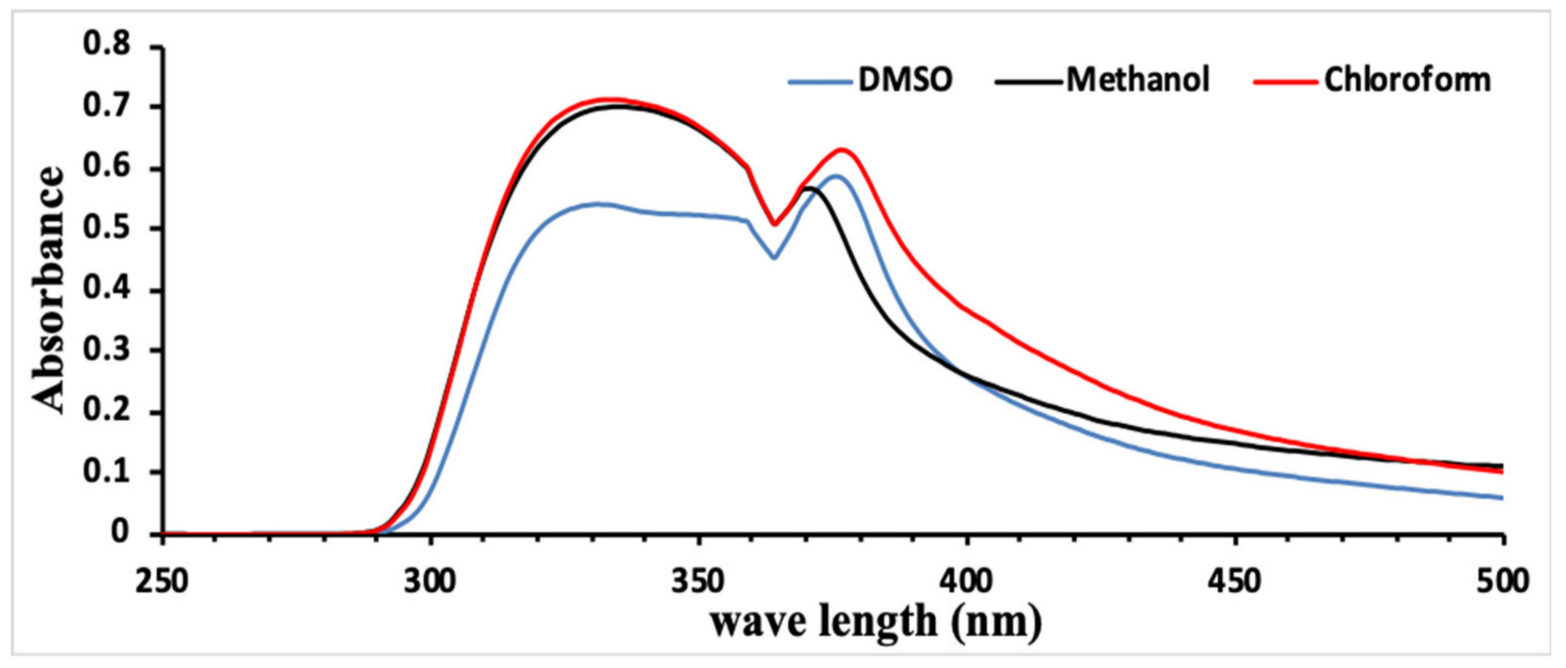
UV-Visible spectra of 1 × 10^−5^ M of Compound **3** in methanol (black line), chloroform (red line), and DMSO (blue) at room temperature.

The UV-Visible absorption spectra of Compound 3 were recorded in the ranges of 250–500 nm in various solvents like CHCl_3_, DMSO, and methanol at room temperature, as reported in Table 6 and shown in Figure 3 Compound **3** showed two intense peaks in CHCl_3_ and methanol solvents, which are due to the possible existence of this compound in tautomerism. However, in the case of using DMSO as solvent, three absorption peaks were detected, which indicate the presence of zwitterion together with enol and keto forms. This result is coincidental to that observed during the measurement of this compound in ^1^H-NMR spectroscopy. The first band is located at 330–334 nm and can be assigned to the π-π^*^ transition of C=C enolic form and thus form is favored by less polar solvent. The second band located at 371–378 nm corresponds to a n-π^*^ transition involving the n-electrons of the carbonyl group [56]. The maximum absorption wavelength and the shape of these peaks were different depending on the solvent. The λ_max_ of Compound **3** in a polar protic solvent such as methanol was 334 nm, and in the aprotic polar DMSO solvent, it was 330 nm, whereas the spectrum in the non-polar CHCl_3_ solvent was 333 nm. This means that the absorption maximums are dependent on the solvent. Increasing the solvent polarity causes a shift in the absorption maximum from 330 nm to 334 nm, relative to the enol band, and from 371 nm to 378 nm, relative to the keto band.

The absorption intensity was also solvent-dependent. The first band was more intense than the second one indicating the existence of both keto and enol tautomers. The enol absorption band was predominated by the keto band in the chloroform solvent but the keto absorption band was dominated by enol absorption band in the DMSO solvent (Table 6). In the case of using polar protic methanol as a solvent, it was observed that the band of enol intensity was higher than the keto band. This refers to the formation of the ion pair in the polar protic of solvent that is involved in hydrogen bonding with the solvent, making it less available to the hydrogen bond with the keto form. The maximum absorption wavelength, intensity, and shape of the bands in spectrum of Compound **3** were different, depending on the solvent polarity. The change of the solvent polarity caused the change in the hydrogen bonding, leading to the change in the tautomerism. Thus, the intensity of absorption of the enol tautomer band decreased, while the absorption of the band of the keto tautomer increased by changing the solvent from nonpolar to polar solvent. This confirms that Compound **3** is present as the keto-enol equilibrium, depending on the polarity of the solvent. On the basis of the existence of an equilibrium between the keto and enol forms, the ultraviolet spectrum of Compound **3**, with respect to the change in the solvent polarity, is found to be difficult to determine as regards the shifting character of the equilibrium percentage towards the keto or enol tautomer forms in organic solvent. The result is that the proportional percentages of the keto and enol forms were not able to be measured quantitatively from the absorption bands, and this was due to the overlapping of these two bands in an individual solvent [57].

#### 3.1.6. Mechanism of Keto-Enol Tautomer Forms

**Scheme 5:**
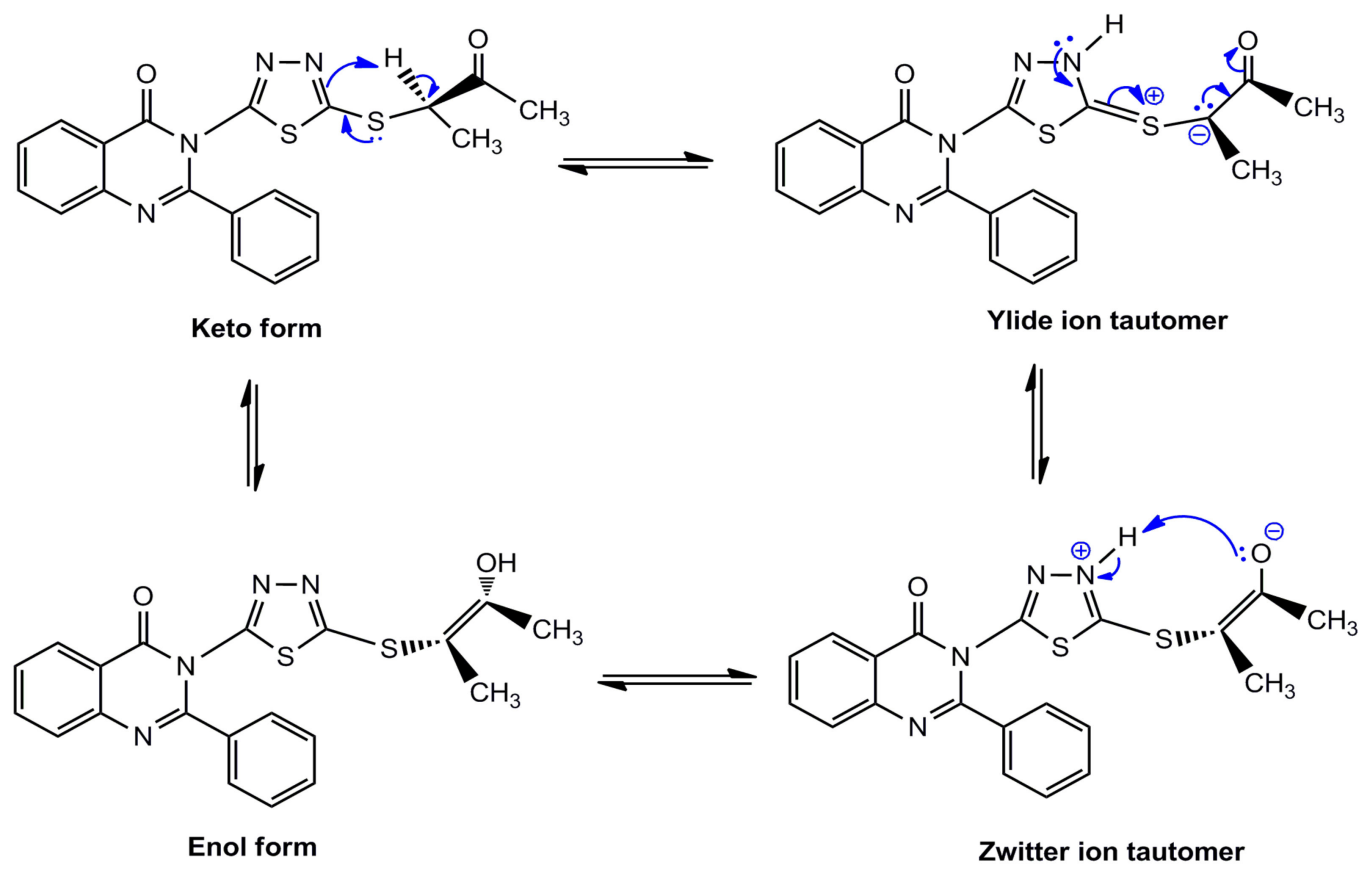
Mechanism of keto-enol tautomer isomers of Compound **3.**

Compound **3** undergoes keto-enol tautomerization on the basis of the spectroscopy data. The mechanism of this process occurs because of the α-hydrogen that is adjacent to carbonyl and because the sulfur atom is slightly acidic. The nitrogen atom of the thiadiazole ring behaves as a strong base, due to the resonance through its conjugation with the sulfur atom, and this base abstracts the hydrogen from carbon to nitrogen, probably via intra-molecular hydrogen bonding, to form a ylide ion tautomer which has a stable structure, by either the electron withdrawing from the sulfonyl cation group or due to the delocalization of the charge into the carbonyl group. The stability of this ion is enhanced by the delocalization of the charge in the heterocyclic ring. The subsequent rearrangement of the ylide ion may generate the zwitterion form by transferring the negative charge to the oxygen atom. However, the formation of a strong intra-molecular hydrogen bond between zwitterion and the N-H group leads to the formation of the cis-enol tautomer. The cis-enol form is stabilized by a strong intra-molecular hydrogen bond, which is present in the methyl group at α-position carbon with its double bond character. The position of the keto-enol equilibrium strongly depends on the electronic character and nature of the solvent. Thus, Compound **3** is obtained as keto-enol tautomeric forms through the zwitterion form with the catonic iminium nitrogen of the thiadiazole unit and enolate groups (Scheme 5).

## 4. Conclusions

In conclusion, the molecular structure and spectroscopic properties of the title compound were synthesized and characterized by FTIR, ^1^H-NMR, ^13^C-NMR, mass, and UV-Visible spectroscopy. The FTIR spectrum revealed the existence of the keto-enol form with two types of intra-molecular hydrogen bonding in the solid state. The^1^H-NMR and UV-visible spectra were recorded in different organic solvents. The results showed that the keto/enol percentage of Compound **3** increases with the increasing solvent polarity. These results identified that the strength of an intramolecular H-bonding was a key factor in controlling the enol-keto equilibrium. In addition, the results of ^13^C-NMR and mass spectrum confirmed that Compound **3** exists in the keto-enol form.

## Figures and Tables

**Table 1:** Selected ^1^H-NMR chemical shifts for Compound **3** in DMSO-*d_6_*, CDCl_3_ and CD_3_OD solvents.

Solvent.	Chemical Shift in ppm	Assignment
CDCl_3_	4.46–4.51 (q, 1H, -CH-)	keto tautomer
	11.71 (s, 1H, OH)	Enol tautomer
DMSO-*d_6_*	4.47–4.52 (q, 1H, -CH-)	keto tautomer
	10.95 (s, 1H, OH)	Enol tautomer
	13.26 (s, 1H, ^+^N-H)	Zwitter ion
CD_3_OD	4.41–4.45 (s, 1H, -CH-)	Only peak of keto form is observed due exchangeable of OH proton by deuterated methanol solvent

**Table 2:** The keto-enol percentage and equilibrium constant of Compound **3** in different solvent polarities.

Solvent	Enol %	Keto %	ZwitterIon	KC
CDCl_3_	59.3	40.7	-----------------	1.46
DMSO-*d_6_*	19.3	61.4	19.3	0.31
CD_3_OD	---------	100	---------------	--------------

**Table 3:** ^13^C-NMR chemical shifts for compound **3** in DMSO-*d_6_*.

Carbon	Chemical Shift
CH, 7 x aromatic CH	122.9, 123.5, 127.4, 128.7, 129.5, 132, 132.8
C, 3 x aromatic C	134.4
C, 2 x C=N	137.9
C enol, C quinazolinone ring	123.9
C=C-OH enol	155.5
C, CO lactam	165.1
C, CO -CO-CH_3_	204.5

**Table 4:** The selected molecular ions with their formula and their relative abundance of Compound **3**.

Molecular Ions	Relative Abundance	Formula
161	28	C_5_H_7_NOS_2_^+^
146	25.6	C_4_H_4_NOS_2_^+^
60	6.4	C_2_H_4_S^+^ Keto Tautomer
43	35.2	C_2_H_3_O^+^
407	4.8	C_20_H_15_N_4_O_2_S_2_^+^
224	100	C_13_H_10_N_3_O^+^
203	20.8	C_9_H_5_N_3_OS^+^
179	12	C_10_H_11_OS^+^ Enol Tautomer
120	16.8	C_8_H_8_O^+^
105	87.2	C_7_H_5_O^+^
77	64	C_6_H_5_^+^

**Table 5:** The selected molecular ions with their formula and their relative abundance of Compound **1**.

Molecular Ions	Relative Abundance	Formula
203	75	C_6_H_9_N_3_OS_2_^+^
161	100	C_5_H_7_NOS_2_^+^
146	30	C_4_H_4_NOS_2_^+^
128	85	C_4_H_2_NS_2_^+^
60	48.75	C_2_H_4_S^+^
43	63.75	C_2_H_3_O^+^

**Table 6:** UV absorption bands, molar extinction coefficient (L.mol^−1^.cm^−1^), and solvent effects on the tautomerism of Compound **3** in different solvents at room temperature.

Solvent	Enol Form			Keto Form			Zwitterion Form		
	λmax (nm)	Abs	ε	λmax (nm)	Abs	ε	λmax (nm)	Abs	ε
Methanol	334	0.702	70,200	371	0.589	58,900	-	-	-
Chloroform	333	0.712	71,200	378	0.625	62,500	-	-	-
DMSO	330	0.541	54,100	373	0.609	60,900	345	0.526	52,600
